# Medical education during the COVID-19: a Malaysian experience

**DOI:** 10.5116/ijme.6231.a20e

**Published:** 2022-03-31

**Authors:** Fairrul Kadir, Boon Tat Yeap, Firdaus Hayati, Fatimah Ahmedy, Farhana Harzila Mohd Bahar, Mohammad Saffree Jeffree

**Affiliations:** 1 Emergency Medicine Department, Faculty of Medicine and Health Sciences, Universiti Malaysia Sabah, Kota Kinabalu, Malaysia; 2 Anaesthesia and Intensive Care Department, Faculty of Medicine and Health Sciences, Universiti Malaysia Sabah, Kota Kinabalu, Malaysia; 3 Surgery Department, Faculty of Medicine and Health Sciences, Universiti Malaysia Sabah, Kota Kinabalu, Malaysia; 4 Medical Education Department, Faculty of Medicine and Health Sciences, Universiti Malaysia Sabah, Kota Kinabalu, Malaysia; 5 Public Health Medicine Department, Faculty of Medicine and Health Sciences, Universiti Malaysia Sabah, Kota Kinabalu, Malaysia

**Keywords:** Medical education, COVID-19, Malaysian experience

## Introduction

The novel coronavirus (SAR-CoV-2) pandemic has affected all levels of education, including medical education worldwide.^1^ Most medical schools have had to withhold all face-to-face classes and resort to online classes, adapting from live clinical exposure to a virtual one. This conversion to online methods has led to many difficulties in delivering psychomotor and affective topics, thus leaving many concerns on the quality of graduates produced. However, the pandemic has enhanced online education, telehealth, and adaptive research protocol advancement in another perspective. These occurred as all parties embarked on exploration for the best online class and examination approach to surrogate face to face exposure.^2^^,^^3^ This article discussed the experience of the medical students in the Medical Doctor Programme of the Universiti Malaysia Sabah (UMS) in adapting the medical students' training with the emergence of the coronavirus disease (COVID-19) pandemic.

### Conversion to Online Classes

In the wake of the pandemic, the university has converted all teaching and learning activities online. Similarly, the Medical Doctor Programme of UMS was no exception. Online learning, which is a part of e-learning, is a teaching and learning assisted by the application of technology.^4^ It has presently been applied extensively in all levels of education, including medical education, which has been acknowledged by the World Health Organisation as a helpful tool in addressing the educational needs of healthcare workers.^5^^,^^6^ However, the faculty has experienced using online methods only to support face to face classes, mainly in preclinical years. The clinical classes have always been done bedside conventionally and in a healthcare facility setting. Student and lecturer perceived lecturer-centred teaching as the best mode as the lecturer systematically simplifies the complex concepts, which enhances further analysis, interpretation, and evaluation in all teaching domains.^7^

When the idea for an extended online academic delivery was suggested, there was a mixed response among the faculty members. Some lecturers and supporting staff were against the idea and proposed postponing the academic calendar while waiting for conditions to become safer. After in-depth considerations on the uncertainty of the pandemic's duration and the possible complications of backlogged students, the faculty decided to explore new online methods to ensure the continuity of medical education. This approach was supported by previous experiences of other medical schools in China and Korea that had gone through the Severe Acute Respiratory Syndrome (SARS) and the Middle East respiratory syndrome (MRES) outbreaks with innovative approaches.^8^^,^^9^

### Amendments in Academic Policies

Firstly, the faculty needed to determine the priorities for the classes and examinations. The faculty divided the student batches into high stake batches and low stake batches. Year three and year five were identified as high-stake batches. There are professional examinations for these two batches, and year five complete their final year before graduating as a full-fledged doctor. The remaining batches were classified as low stakes and viewed as having ample time for revisions and improvement before graduation. This division is essential to determine the extent to which the classes and examinations can be done virtually online and channel resources appropriately.

Secondly, the faculty had to ensure the continuity of the academic calendar. As the pandemic and lockdowns were very dynamic, the academic calendar had to be flexible for any sudden changes. For this, the faculty divided each rotation into theory and clinical/practical components. The theory components were delivered online during the lockdowns, while the clinical/practical components were conducted face to face in the faculty/hospital whenever the condition was safe. It is interesting to mention that the academic calendar for UMS Medical Doctor Programme was amended twenty-four times during 2019/2020.

### Initiation of Online Teaching and Learning

The mode of the online class was selected based on their teaching domains. Classes with a cognitive background such as lectures, Small Group Discussions, Problem-based Learning and seminars were conducted using Google Meet, Cisco Webex, Zoom, and UMS's own Learning Management System, smart2UMS. The tricky part was to deliver classes involving psychomotor and affective domains such as Bedside Teaching, Ward Rounds, Practical and Clinical Skills Lab online. Besides, it was difficult to obtain actual patients for clinical exposure during online clinical classes. The only method available was to conduct the virtual classes synchronously with the aid of simulations such as scenarios, pictures, sound recordings and demonstrations using mannequins. However, limited literature supports psychomotor and affective skills delivery through the online learning method. Cooper and Higgins evaluated the effectiveness of online instructional videos for acquiring and demonstrating cognitive, affective, and psychomotor skills amongst undergraduate students.^10^ The study revealed that it is a medium likely to benefit a proportion of a cohort, which may be harmful to students' learning. However, conclusive evidence for their usage has not been established and requires further research.^10^ Therefore, high stake years had called for face to face classes whenever possible in between the lockdown periods, while low stake years continued with online classes until the lockdowns had been fully lifted.

A schedule for online classes was made, and students were contacted to attend their respective classes through the WhatsApp and Telegram applications. Furthermore, an Online Academic Support Group consisting of experienced staff mainly from the Medical Education Department, e-Learning Unit, and Academic Unit was set up to support the faculty's implementation of online academic activities. This group organised regular orientations and hands-on workshops for lecturers and students to familiarise themselves with the available online platforms. In addition, guidelines on performing online academic activities and training videos were prepared for the faculty members.

While making all these plans, the faculty also ensured that the changes aligned with any regulations issued by the local medical education authority. The Malaysian Medical Council Regarding issued regulations, a guide to Operating Medical Education Program (Basic Degree) During and Post COVID-19 Movement Control Order which states classes involving Miller's Pyramid of Clinical Competence level 3 (show how) and level 4 (does) is given the flexibility to conduct a virtual synchronous class with the condition that it is conducted synchronously.^11^^,^^12^

### Completing the Process with Online Assessments and Evaluations

The real challenges were conducting assessments and evaluations during the pandemic and lockdowns. Any changes in the assessments and evaluations must not compromise the examinations' fairness, justice, and integrity. Numerous simulations were conducted to postpone all examinations on the academic calendar. However, the results revealed that the examinations would be tightly clumped together, leading to reduced faculty resources and intense pressure on the students. In addition, postponing all examinations would lead to student batches being unable to proceed to the subsequent semester, as it is a prerequisite to passing each semester before moving to a new one. Therefore, it was inevitable for examinations in the low stake years to be converted online while making way for high stake year examinations to be postponed until a safer time for face to face activities was obtained.

To convert the examinations to an online format, the faculty had to look back thoroughly into the programme and course learning outcomes ensuring that they matched with each examination. This is important to ensure the online versions of the examinations would measure students in the correct topics, domain, and taxonomy to fulfil the requirements of Outcome-Based Education. In addition, the faculty also identified a list of clinical competencies required by medical students to help them choose alternative examination methods accurately.

In the conventional format, UMS practices summative assessment is divided into theory and clinical examinations. Excluding the continuous assessments, the theory examination consists of Multiple-Choice Questions (MCQ), Modified Exam Questions (MEQ) and Long Essay Questions (LEQ). In contrast, the clinical examinations include Long Case, Short Case, Objective Structured Clinical Examinations (OSCE) and Objective Structured Practical Examinations (OSPE). Each of these existing examination formats was categorised based on their domains and competencies before being matched up with an appropriate online examination format. Competencies were categorised into applying knowledge, problem-solving, clinical reasoning, ethical reasoning, diagnostic ability, patient management, history taking skills, communication skills, physical examination, and professionalism. In contrast, domains were divided into cognitive, psychomotor, and affective.

From these classifications, theory examinations within the cognitive domain were easily converted into online formats in the smart2UMS, as the platform already had the required features. However, the tricky part was how to ensure the integrity and fairness of the online examinations. For this, the faculty adopted the online proctored examination method, where students were required to have two devices; one for performing the examination and another one that acts as a camera to monitor students and their surroundings during the examination. At the same time, each student was required to sign an integrity declaration letter.

Meanwhile, examinations were done synchronously online and via pre-recorded videos for the clinical examinations, which falls under psychomotor and affective domains. Clinical questions were derived from actual patients and simulated patients with the aid of pictures, diagrams, and sound recordings. Questions involving skills such as physical examinations and procedures required students to perform it on friends, family members or mannequins. Examiners will give feedback on students' performance, and regularly, students were required to repeat their session or resubmit another video until their performance was deemed satisfactory. All virtual synchronous examinations were conducted using Google Meet.

On the other hand, the faculty still understood that online clinical classes and examinations could never replace a face to face and hands-on approach. Therefore, these online methods were made only as a temporary measure and focused on the low stake batches only. It was hoped that the batches that had to go through these online approaches would have the chance to improve themselves in the remaining years before they graduate.

To ensure the success of the online examinations, all parties, including the lecturers, support staff and students, needed to be familiar with these new methods. Thus, online mock examinations were conducted at least twice for the low stake year students involved. These mock exams would later prove beneficial as they improved the familiarisation of the system and was an excellent platform to test the logistic capabilities of all sides, especially students. In addition, students who found that their internet connections or hardware were not good enough for synchronised examinations had ample time to make necessary changes such as borrowing laptops and moving in with relatives with better internet connections during the examination period. The Online Academic Support Group also played an extensive role in supporting the implementation of these online examinations. This group was responsible for assisting the lecturers in online theory examinations using the smart2UMS platform, setting up online 'examination rooms' for clinical examinations, and coordinating interchange between examiners and students. They also looked into improving the internet services in the faculty and attending to any troubleshooting.

Another adjustment was converting all theory examinations into MCQ. This adjustment was made because it is not practical to type essay questions during a synchronous examination. On the other hand, there were suggestions for an open book and open web examinations with higher cognitive level questions. However, this concept was still new to the faculty, and there was limited time for adaptation. Also, through the smart2UMS platform, MCQ questions and answers arrangements were randomised to prevent students from cheating.

There was a mixed performance of students' online examination results compared to face to face examinations. Online theory examinations generally showed better results than previous cohorts done conventionally. On the other hand, the clinical examination results were relatively similar between the online synchronous and conventional examinations. This outcome appears to be identical to two studies among psychology students in the US, where students performed better in non-proctored online examinations.^13^^,^^14^ Even though the online theory examinations were proctored by examiners using a second device, the possibility of cheating could not be eliminated. Another reason could be that students were less anxious when the examiners were not in the same room during the examinations.

Another critical aspect of these online classes and examination implementation was the continuous quality improvement system. The faculty's Academic Quality Assurance Unit (ACAU) played a pivotal role in ensuring all online academic activities were done following university policies and professional bodies' guidelines (Malaysian Medical Council and Malaysian Qualification Agency). ACAU also monitored the examination process throughout the process to ensure the quality was up to the standards. This includes regular checks on the quality of questions, online vetting sessions, safekeeping of the online question bank, the conduct of proctored online examinations, and examination result analysis.

Feedback from students was actively collected and considered for quality improvements. Overall, students were satisfied with the online classes and examinations conducted with few comments on individual lecturers' online teaching literacy and problems involving poor internet connections. However, students felt that face to face classes were better than online classes. This finding was similar to a study done in an Indian medical school, where it was presumed due to the faculty's inadequate preparations for the new teaching approach.15 In the context of UMS, this response is easily explainable since face to face classes and the examinations provide better interactions for expressions and comprehensions.

## Conclusions

The novelty of this effort lies in the rapid adaptation and implementation of these extensive changes by the whole faculty, including management, lecturers, and students. Categorising between high stake and low stake years also gave the faculty a chance to prioritise efforts in light of limited experience and resources. In addition, the preponement of theoretical classes and postponement of clinical classes, even though it has its own risk and disadvantages, allowed the academic calendar to continue despite the lockdowns. At the same time, the categorisation of competencies required by medical students during their clinical years enabled the faculty to convert the examinations and evaluations to appropriate alternative methods while maintaining the quality. It is believed that this experience can serve as a reference for the faculty and other educational institutions in the future to continue medical education when faced with conditions that disrupt conventional teaching.

### Acknowledgements

We would like to acknowledge and thank the Medical Education Department and Information & Technology Unit of the Faculty of Medicine and Health Sciences, Universiti Malaysia Sabah, for providing information relevant to this writing.

### Conflicts of Interest

The authors declare that they have no conflict of interest.
